# Production of RPSL_12_-TetR fusion and development of two multiplex methods for simultaneous detection of tetracyclines and aminoglycosides in pork

**DOI:** 10.1016/j.fochx.2025.103324

**Published:** 2025-11-27

**Authors:** Wanqiu Xia, Di Zhang, Kuijing Liang, Jiajia Hu, Jiaxuan Chang, Jianping Wang

**Affiliations:** a College of Life Science, Hengshui University, Hengshui, Hebei 053000, China; b College of Veterinary Medicine, Hebei Agricultural University, Baoding, Hebei 071000, China

**Keywords:** Aminoglycosides, Multiplex immunoassays, Pork samples, RPSL_12_, TetR, Tetracyclines

## Abstract

Nowadays, multiplex immunoassays capable of simultaneously detecting two classes of drugs are highly desirable. In this study, the genes of TetR and RPSL_12_ were fused and expressed to produce a novel fusion protein that recognizes 10 tetracyclines (TCs) and 12 aminoglycosides (AGs) simultaneously. A multiplex direct competition fluorescence assay and a multiplex direct competition chemiluminescence assay were established for the simultaneous detection of these two drug classes in pork samples. The multiplex direct competition fluorescence assay required only a single sample addition step and could be completed within 35 min, with detection limits ranging from 1.1 to 10.2 ng/mL. The multiplex direct competition chemiluminescence assay could be completed within 30 min and achieved significantly lower detection limits (0.0016–1.8 ng/mL). Based on a comprehensive evaluation of assay time, operational complexity, and sensitivity, both methods demonstrated superior performance compared to previously reported immunoassays for these drug classes.

## Highlights

• Novel fusion protein enables dual recognition of TCs and AGs.
• First multiplex CL and FL assay developed for TCs and AGs detection.
• Achieves ultra-low LODs (0.0016–1.8 ng/mL) by chemiluminescence.
• Methods greatly reduce reagent use, labor, and analysis time.

## Chemical compounds studied

Tetracycline (CID: 54675776)

Oxytetracycline (CID: 54675779)

Doxycycline (CID: 54671203)

Chlortetracycline (CID: 54675777)

Minocycline (CID: 54675783)

Streptomycin (CID: 19649)

Gentamicin (CID: 3467)

Neomycin (CID: 8378)

Kanamycin (CID: 6032)

Amikacin (CID: 37768).

## 1 Introduction

Over the past several decades, immunoassays have been widely employed as rapid screening tools for detecting numerous contaminants in food and environmental samples (Li et al., 2017a). Commonly used immunoassays include enzyme-linked immunosorbent assay (ELISA), fluoroimmunoassay (FIA), fluorescence polarization immunoassay (FPIA), time-resolved fluoroimmunoassay, fluorescence resonance energy transfer immunoassay, chemiluminescence immunoassay, chemiluminescence resonance energy transfer immunoassay, immunochromatographic strip, and biosensors. However, conventional immunoassays typically detect a single analyte or a single class of analytes. Thus, an immunoassay capable of simultaneously determining two or more classes of analytes is highly desirable. In recent years, novel dual-analyte immunoassays capable of simultaneously detecting two classes of analytes have been reported, including dual-label quantum dot-based immunoassay (Le, Zhu, & Yu, 2016), dual-wavelength fluorescence polarization immunoassay (Zhang et al., 2018), and dual-labeled time-resolved fluoroimmunoassay (Sheng et al., 2016; Li et al., 2017).

The immunoassays mentioned above all utilize antibodies—including monoclonal, polyclonal, and recombinant antibodies—as recognition reagents. It is well known that conventional antibody production requires the use of experimental animals, involves tedious procedures, and incurs high costs. Consequently, alternative recognition materials with specific binding capabilities, such as molecularly imprinted polymer (Chen et al., 2018), have been developed for use in pseudo-immunoassays. For instance, in our recent study, molecularly imprinted microspheres served as recognition reagents to develop a multiplexed fluorescence method for the simultaneous detection of benzimidazoles and pyrethroids (Cai et al., 2020). Nonetheless, all these recognition reagents are derived from a single antibody or template molecule, and their recognition capabilities can vary significantly. Therefore, it is necessary to find new recognition reagents.

To further expand the landscape of multi-residue detection methods, recent developments in spectrophotometric techniques have shown promise. In particular, sustainable and miniaturized strategies such as batch spectrophotometry and cloud point extraction have demonstrated applicability for multi-analyte determinations in drug analysis (Albarznji, Hussein, Shabaa, Abbadi, & Azooz, 2025). These advances complement receptor -based platforms and offer greener alternatives. As naturally occurring proteins, receptors can be expressed recombinantly in a process that is simpler, faster, and more cost-effective than antibody production. Accordingly, several receptor-based pseudo-immunoassays have been reported for various analytes (Ahmed et al., 2017; Santillo, 2020), including sulfonamides (He, Cui, Liu, Feng, & Wang, 2022), β-lactams (Ahmed et al., 2020), and estradiol (Busayapongchai & Siri, 2017). These studies demonstrate that the performance of receptor-based methods is generally superior to that of antibody-based immunoassays. However, all reported receptor-based methods are limited to the detection of a single drug class. A receptor-based method capable of simultaneously determining two or more drug classes has not yet been reported.

Tetracyclines and aminoglycosides are broad-spectrum antibiotics commonly used to treat bacterial infections in food-producing animals. Residual amounts of these drugs in animal-derived foods may pose health risks to consumers, driving the development of numerous methods for detecting their residues in various food matrices (Pérez-Rodríguez, Pellerano, Pezza, & Pezza, 2018). Among the reported methods, some immunoassays can detect multiple TCs (Hu et al., 2024; Xing et al., 2024), whereas most AG immunoassays are limited to detecting a maximum of two drugs (Jiang et al., 2018a; Wei, Meng, Zeng, & Huang, 2019; Li et al., 2017b). Recently, several receptor-based pseudo-immunoassays have been reported for detecting TCs (Meyer, Chatelle, Weber, Niessner, & Seidel, 2020; Wang, Xia, Liu, Wang, & Liu, 2019; Wang, Zhang, Liu, & Wang, 2019) and AGs (Xia, Liu, & Wang, 2022). For example, in our recent study, the TetR of *Escherichia coli* is produced that is used as receptor to develop a chemiluminescence method for the detection of 10 TCs (Xia, Cui, Wang, & Liu, 2021). In another study, we produced the RPSL_12_ from *Escherichia coli* and utilized it as a receptor to develop a fluorescence polarization method for detecting 10 AGs (Xia, Zhang, & Wang, 2022).

Despite these advances, no existing method can simultaneously detect both TCs and AGs. The recognition mechanisms of the RPSL_12_ and TetR receptors were elucidated in our previous work through homology modeling and molecular docking. (Xia et al., 2021; Xia, Zhang, & Wang, 2022). In this study, the TetR and RPSL_12_ genes were linked and expressed to produce a novel fusion protein, RPSL_12_-TetR. Furthermore, two enzyme-labeled conjugates and two fluorescent tracers were synthesized. These reagents were used to develop multiplex chemiluminescence and fluorescence immunoassays in conventional microplates for the simultaneous detection of TCs and AGs.

**Fig. 1:**
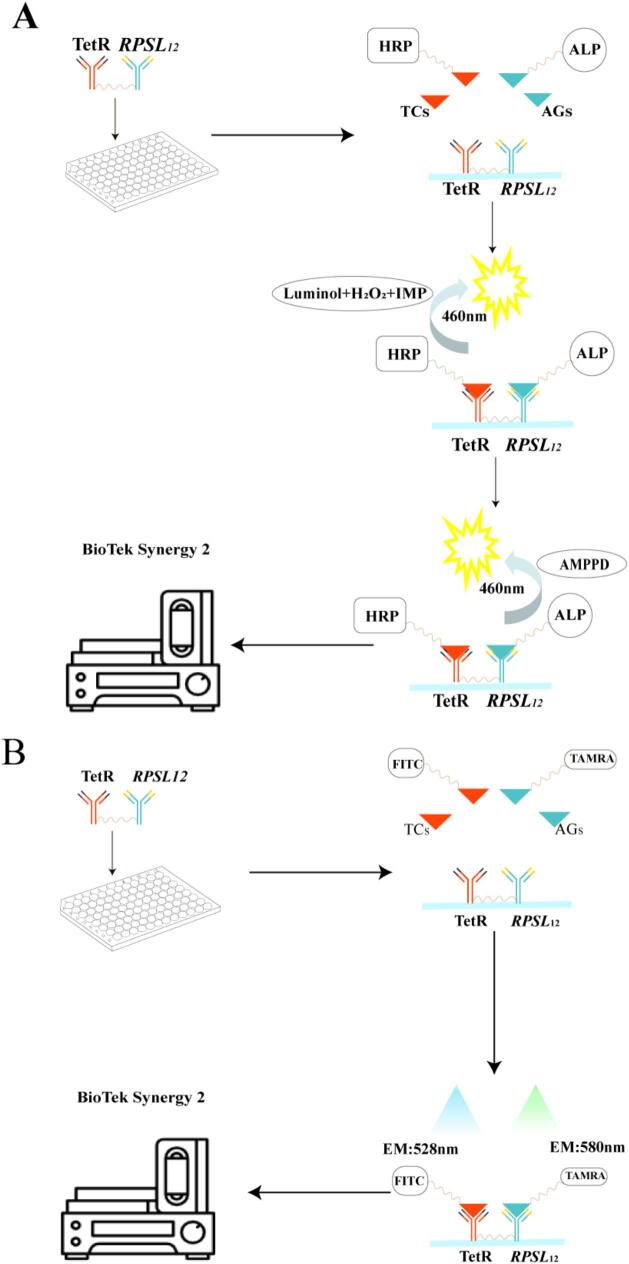
(A) Schematic diagram of the developed multiplex direct competition chemiluminescence assay. (B) Schematic diagram of the developed multiplex direct competition fluorescence assay.

As illustrated in Fig. 1A, RPSL_12_- TetR was coated onto microplate wells. Subsequently, a sample extract and two enzyme-labeled conjugates were added for direct competitive binding. Following this, two distinct luminescent substrates were added sequentially, and chemiluminescence signals at specific wavelengths were measured to quantify TCs and AGs independently. As shown in Fig. 1B, an alternative format involved incubating RPSL_12_-TetR-coated wells with a sample extract and two fluorescent tracers. Fluorescence signals were then recorded at distinct excitation/emission wavelength pairs to independently quantify the two drug classes. After optimizing key assay parameters, both methods were successfully applied to the simultaneous determination of TCs and AGs residues in pork samples.

## 2 Materials and methods

### 2.1 Chemicals

The standards of streptomycin (STR), neomycin (NEO), gentamicin (GEN), amikacin (AMK), spectinomycin (SPM), apramycin (APM), paromomycin (PMM), netilmicin (NTM), Isepamicin (ISM), Sancycline (SC), Kanamycin (KAN), Luminol, 3-[2-spiroadamatane]-4-methoxy-4-[3-phosphoryloxy]-phenyl-1,2-dioxetane) Dioxetane (AMPPD), 6-carboxytetramethylrhodamine(TAMRA) and fluorescein isothiocyanate (FITC) were from Shanghai Yuanye Biological Technology Co., Ltd. (Shanghai, China). The standards of micronomicin (MIM) and etimicin (ETM) were from China National Institute for Food and Drug Control (Beijing, China). The standards of tetracycline (TC), oxytetracycline (OTC), doxycycline (DC), tigecycline (TIC), chlortetracycline (CTC), demeclocycline (DMC), minocycline (MC), horseradishperoxidase (HRP), N-hydroxysuccinimide (NHS), 4-(imidazol-1-yl) phenol (IMP) and 1-ethyl-3-(3-dimethylaminopropyl)-carbodiimidehydrochlorde (EDC) were from Sigma-Aldrich (St.Louis, USA). Methacycline (MTC) was from J&K Scientific Ltd. (Beijing, China). Lymecycline (LMC) was from Toronto Research Chemicals (Toronto, Canada). Alkaline phosphatase (ALP) was from Maclean Biochemical Technology Co (Shanghai, China).

### 2.2 Expression of RPSL_12_-TetR fusion

The recombinant fusion protein RPSL_12_-TetR was expressed and purified. The RPSL_12_ gene from *Escherichia coli* (GenBank accession no. CAD6001255.1) and the TetR gene from Enterobacteriaceae (GenBank accession no. WP_060614418.1) were ligated and cloned into the expression vector pET-32a (+) by Sangon Biotech. The recombinant plasmid (1 μL) was mixed with 100 μL of competent cellsand incubated on ice for 30 min. Heat shock was performed at 42 °C for 90 s, followed by immediate incubation on ice for 5 min. Then, 500 μL of Luria-Bertani (LB) broth was added, and the cells were recovered by shaking at 37 °C and 220 rpm for 1.5 h. A 50 μL aliquot of the culture was spread onto LB agar plates and incubated overnight at 37 °C. A single positive colony was selected to inoculate 10 mL of LB broth. When the OD600 reached 0.6–0.8, protein expression was induced with 0.5 mM Isopropyl β-D-1-thiogalactopyranoside (IPTG), and the culture was incubated at 22 °C with shaking at 180 rpm. After induction, the bacterial culture was centrifuged at 8000 rpm for 10 min at 4 °C. The pellet was resuspended in 10 mL of phosphate-buffered saline (PBS, pH 7.4) and lysed via ultrasonication on ice for 15 min. The lysate was centrifuged at 8000 rpm for 10 min at 4 °C, and the supernatant, containing the soluble target protein, was purified using Nickel-Nitrilotriacetic acid (Ni-NTA) agarose affinity chromatography. The purified RPSL_12_-TetR fusion protein was characterized by sodium dodecyl sulfate–polyacrylamide gel electrophoresis (SDS-PAGE) and western blot analysis. (Wang, Xia, et al., 2019).

### 2.3 Synthesis of two enzyme-labeled conjugates

The hapten for MC, synthesized in our previous study by coupling with 4-aminobenzoic acid (Xia et al., 2021), was conjugated with horseradish peroxidase (HRP) to prepare the MC-HRP tracer. Briefly, 11.8 mg of the MC hapten was dissolved in 3 mL of Dimethylformamide (DMF). Then, 15.3 mg EDC and 9.2 mg of NHS were added, and the mixture was stirred at 4 °C for 4 h (Solution A). Separately, 20 mg of HRP was dissolved in 3 mL of PBS (0.01 M, pH 7.4) (Solution B). Solution A was added dropwise to Solution B, and the resulting mixture was stirred at 4 °C overnight. The conjugate was purified by dialysis against PBS (0.01 M, pH 7.4) at 4 °C for 3 days. The final MC-HRP conjugate was aliquoted and stored at −20 °C until use.

The STR-ALP conjugate was prepared by linking STR to ALP using glutaraldehyde. Specifically, 5.8 mg of STR was dissolved in 1 mL of deionized water (Solution A), and 12.5 mg of ALP was dissolved in 3 mL of PBS (0.01 M, pH 7.4) (Solution B). Solution A was added dropwise to Solution B, followed by the addition of 16 μL of 25 % glutaraldehyde solution. The reaction proceeded at 4 °C for 6 h with gentle stirring. The STR-ALP conjugate was subsequently purified by dialysis against PBS and stored in aliquots at −20 °C.

### 2.4 Synthesis of two fluorescent tracers

The fluorescent tracer MC-FITC was synthesized by conjugating MC with FITC, adapting a method from a previous study (Xia, Zhang, & Wang, 2022). Briefly, Solution A was prepared by dissolving 12 mg of MC and 25 μL of triethylamine (TEA) in 1 mL of methanol. Solution B was prepared by dissolving 8 mg of FITC in 1 mL of methanol. Solution B was added dropwise to Solution A, and the reaction mixture was stirred at 4 °C for 4 h in the dark. The product was purified by thin-layer chromatography (TLC) using a mobile phase of benzene and methanol (3:1, *v*/v). The target band was scraped from the plate and extracted with methanol (3 × 5 mL). The combined extracts were concentrated under reduced pressure to obtain MC-FITC, which was stored at −20 °C protected from light.

The STR-TAMRA tracer was prepared by conjugating STR with TAMRA. First, TAMRA (11 mg) was activated by dissolving it in 200 μL of DMF containing 19.1 mg of EDC and 11.5 mg of NHS, followed by stirring overnight at room temperature. Separately, 12 mg of STR was dissolved in 3 mL of PBS (0.01 M, pH 7.4). The activated TAMRA solution was added dropwise to the STR solution with stirring at 4 °C for 4 h. The conjugate was purified by TLC with the same mobile phase (benzene:methanol, 3:1, v/v). The target band was extracted with deionized water (3 × 5 mL), and the resulting STR-TAMRA was concentrated under reduced pressure and stored at −20 °C in the dark.

### 2.5 Development of multiplex chemiluminescence assay

The multiplex chemiluminescence assay was developed as follows. Microplate wells were coated with 100 μL of the RPSL_12_-TetR fusion protein diluted in PBS by incubation at 37 °C for 2 h. After blocking with 200 μL of 5 % skim milk in PBS for 30 min at 37 °C and washing three times with PBS, the plates were incubated with a mixture containing 50 μL of standard solution, 50 μL of STR-ALP conjugate, and 50 μL of MC-HRP conjugate at 37 °C for 30 min.

Following another three washing cycles, the chemiluminescence signals for the MC-HRP conjugate were measured first. Specifically, 50 μL each of 5 mM luminol, 1 mM hydrogen peroxide, and 2 mM IMP were added to each well. After a 3-min incubation, the luminescence intensity was recorded at an emission wavelength of 460 nm. The solutions were then removed, and the wells were washed. Subsequently, 50 μL of 1 mM AMPPD was added and incubated for 2 min before measuring the luminescence at 460 nm for the STR-ALP conjugate.

Key experimental parameters, including the coating concentration of the RPSL_12_-TetR fusion protein, the working concentrations of the enzyme conjugates, and the incubation times, were systematically optimized. Dose-response curves were established for the 12 aminoglycosides and 10 tetracyclines. The half-maximal inhibitory concentration(IC_50_) and limit of detection(LOD) values were subsequently determined for each antimicrobial agent through logistic regression analysis.

### 2.6 Development of multiplex fluorescence assay

The multiplex fluorescence assay was developed as follows. Microplate wells were coated with 100 μL of RPSL_12_-TetR fusion protein diluted in PBS by incubation at 37 °C for 2 h. After blocking with 200 μL of 5 % skim milk in PBS for 30 min at 37 °C, the plates were washed three times with PBS. Subsequently, a mixture containing 50 μL of tetracycline standards, 50 μL of aminoglycoside standards, 50 μL of MC-FITC, and 50 μL of STR-TAMRA was added to each well and incubated at 37 °C for 35 min. Following three additional washing cycles, fluorescence measurements were performed using excitation at 485 nm and emission at 528 nm for tetracyclines detection, and excitation at 530 nm with emission at 580 nm for aminoglycosides detection.

Competitive inhibition curves for 12 aminoglycosides and 10 tetracyclines were generated by plotting the FP/FP_0_ ratio (where FP represents fluorescence intensity at each concentration and FP_0_ denotes fluorescence intensity at zero concentration) against the logarithmic drug concentration (Log C). Based on these curves, the IC_50_ and LOD were calculated for each antimicrobial agent.

### 2.7 Sample preparation and method evaluation

The extraction of aminoglycosides and tetracyclines from pork samples was performed as follows. After removing adipose tissues from the pork samples, 2 g of homogenized samples were accurately weighed and transferred into 10 mL centrifuge tubes. Then, 5 mL of 3 % trichloroacetic acid (TCA) solution was added to each tube. Following thorough vortex mixing, the tubes were centrifuged at 8000 rpm for 5 min. This extraction procedure was repeated three times, and the combined supernatants were neutralized to pH 7.0 with 30 % NaOH. Appropriate aliquots of the extracts were taken for subsequent analysis.

To evaluate the method, blank pork samples (pre-confirmed to be free of TC and AG residues) were collected from controlled slaughterhouses. These samples were fortified with standard solutions of both drug classes at different levels to extraction and analysis.

Finally, fifty retail pork muscle samples were randomly collected from multiple local supermarkets and analyzed following the aforementioned analytical procedures.

## 3 Results and discussions

### 3.1 Characterization of RPSL_12_-TetR fusion

**Fig. 2:**
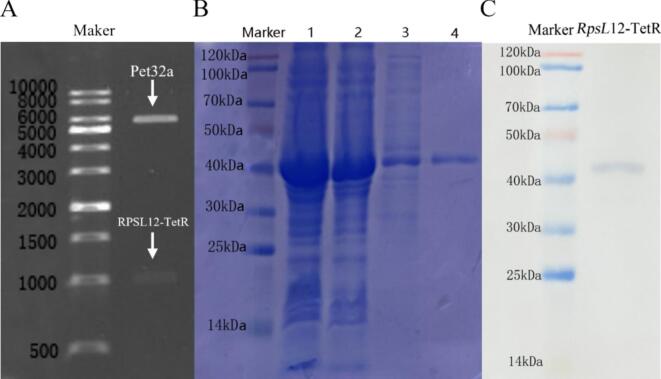
(A) Express vector pET32a-RPSL_12_-TetR after enzyme digestion. (B) SDS-PAGE results of RPSL_12_-TetR (41 kDa) (lane 1, bacterial whole protein; lane 2, inclusion body; lane 3, supernatant; lane 4, purified supernatant). (C) Western blotting analysis of RPSL_12_-TetR.

The fusion receptor RPSL_12_-TetR was successfully prepared for the first time. The genes of RpsL_12_, TetR and the linker were 372 bp, 636 bp and 45 bp in length, respectively (total 1053 bp). As shown in Fig. 2A, agarose gel electrophoresis confirmed the successful enzymatic digestion of the recombinant plasmid, showing bands corresponding to the pET-32a (+) vector (5626 bp) and the insert (1053 bp). SDS-PAGE analysis (Fig. 2B) indicated that RPSL_12_-TetR was expressed in both insoluble inclusion bodies and the soluble supernatant. For convenience, RPSL_12_-TetR was purified from the supernatant and obtained at a final concentration of 0.2 mg/mL. The purified protein exhibited >95 % purity as determined by SDS-PAGE and densitometric analysis, with a batch-to-batch reproducibility of 9 % coefficient of variation(CV). The protein was stored at −80 °C until use. The theoretical molecular weights of RPSL_12_ and TetR were 14.26 kDa and 24.38 kDa respectively. The observed molecular weight of the purified fusion protein was approximately 41 kDa (Fig. 2C), which correlates well with the expected size when including the mass of the His-tag and linker (approx. 2.3 kDa). The identity of the target protein was further confirmed by western blot analysis (Fig. 2C). The purified RPSL_12_-TetR fusion protein was stored at −80 °C and used for all subsequent experiments.

### 3.2 Characterization of two enzyme-labeled conjugates

**Fig. 3:**
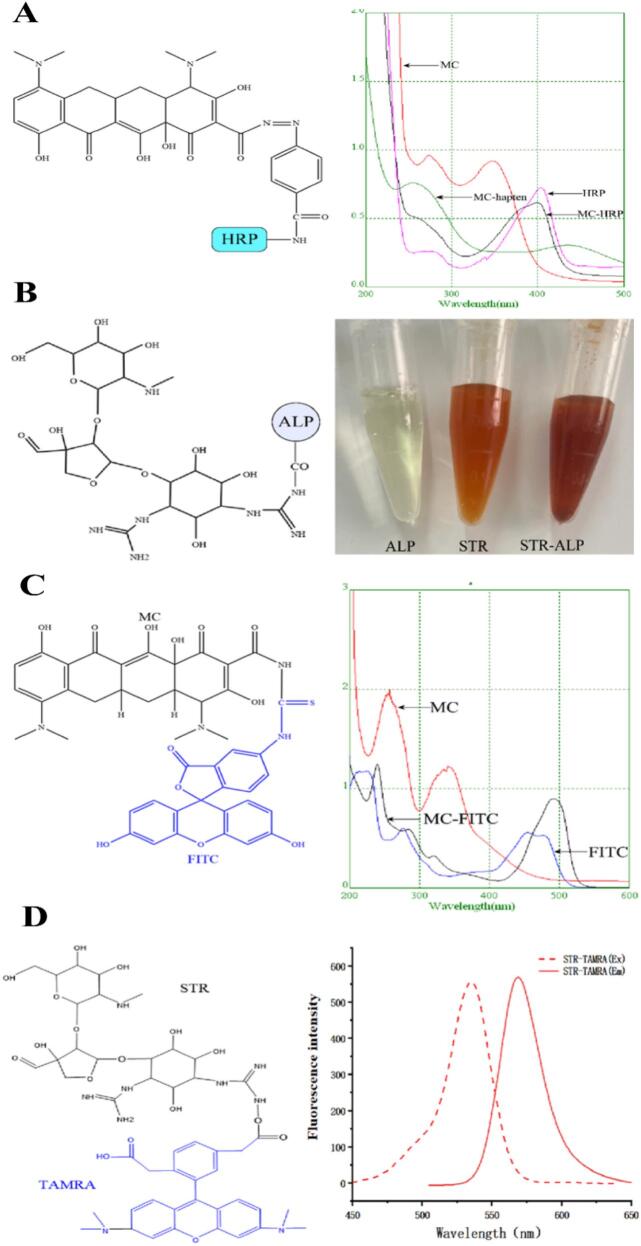
(A) Molecule of MC-HRP, and its UV spectrum. (B) Molecule of STR-ALP, and its maltol reaction. (C) Molecule of MC-FITC, and its UV spectrum. (D) Molecule of STR-TAMRA, and its fluorescencespectrum.

The dual chemiluminescence assay required two enzyme-labeled conjugates. The conjugate (MC-HRP) was synthesized by coupling the MC hapten with HRP. As shown in Fig. 3A, the UV spectrum of the MC-HRP conjugate displayed characteristic absorption peaks of both HRP and the MC hapten, confirming successful conjugation. The STR-ALP conjugate was synthesized from the STR hapten and ALP. Under acidic conditions, STR demonstrates a characteristic maltol-specific chromogenic reaction, which serves as a qualitative indicator for successful enzyme conjugate synthesis. As shown in Fig. 3B, while ALP alone did not react with iron ions, the synthesized STR-ALP conjugate formed a reddish-brown chelate, thereby indicating successful conjugation.

### 3.3 Characterization of two fluorescent tracers

The dual fluorescence assay required two fluorescent tracers. As shown in Fig. 3C, MC was coupled with FITC to obtain the fluorescent tracer (MC-FITC). The UV plot of MC-FITC displayed the characteristic peaks of FITC and the MC, indicating the successful preparation of MC-FITC. As shown in Fig. 3D, STR was coupled with TAMRA to obtain the fluorescent tracer (STR-TAMRA). The fluorescent tracer STR-TAMRA exhibited excitation and emission maxima at 535 nm and 575 nm respectively, indicating the successful preparation of STR- TAMRA.

### 3.4 Optimization of multiplex direct competition chemiluminescence assay

**Fig. 4:**
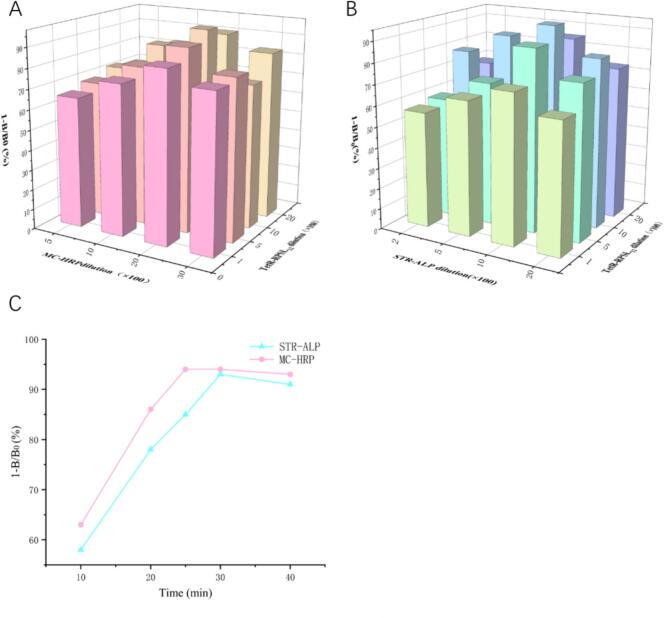
Optimization results for (A) dilution ratios of RPSL_12_-TetR and MC-HRP, (B) dilution ratios of RPSL_12_-TetR and STR-ALP, and (C) incubation time for the chemiluminescence assay.

To enhance sensitivity, several parameters were optimized using STR and TC as representatives. In this study, various dilution ratios of RPSL_12_-TetR, STR-ALP, and MC-HRP were individually mixed with STR and TC (100 ng/mL each) for detection and analysis. As shown in Fig. 4A and B, when the dilution ratios of RPSL_12_-TetR, STR-ALP, and MC-HRP were 1:1000, 1:1000, and 1:2000, respectively, the inhibition rates for STR and TC reached their peak. As shown in Fig. 4C, the inhibition rates for both STR and TC reached their maximum when the incubation time was 30 min. All optimized parameters were applied to subsequent method validation studies.

### 3.5 Optimization of multiplex direct competition fluorescence assay

**Fig. 5:**
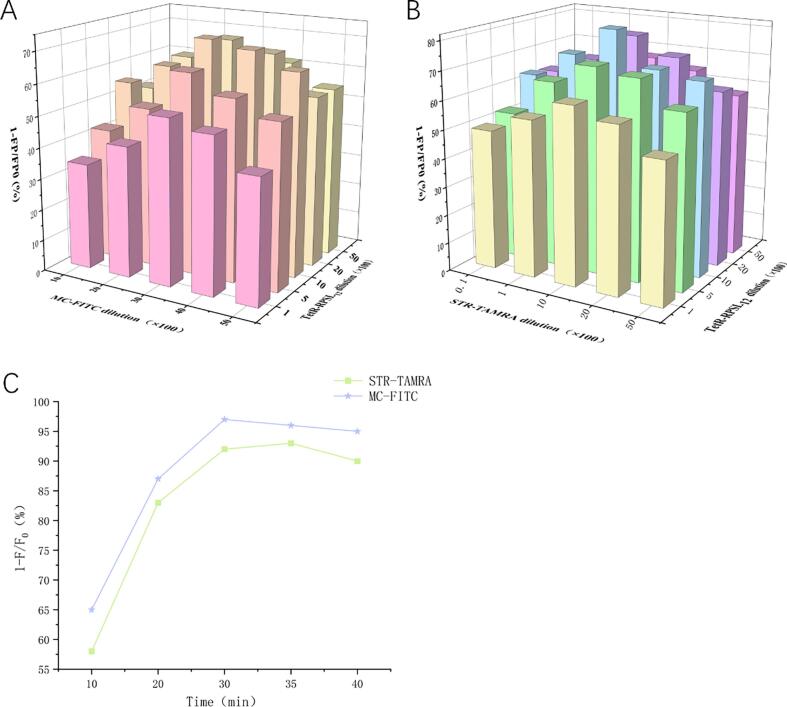
Optimization results for (A) dilution ratios of RPSL_12_-TetR and MC-FITC, (B) dilution ratios of RPSL_12_-TetR and STR-TAMRA, and (C) incubation time for the fluorescence assay.

To enhance sensitivity, several parameters were optimized using STR and TC as representatives. In this study, different dilutions of RPSL_12_-TetR, STR-TAMRA, and MC-FITC were individually mixed with STR and TC (100 ng/mL each) for detection and analysis. As shown in Fig. 5A and B, when the dilution ratios of RPSL_12_-TetR, STR-TAMRA and MC-FITC were 1:1000, 1:1000, and 1:3000, respectively, the inhibition rates for STR and TC reached their peak. As shown in Fig. 5C, the inhibition rates for both STR and TC reached their maximum when the incubation time was 35 min. Therefore, the above optimal parameters are used in the following experiments.

### 3.6 Method performances

**Fig. 6:**
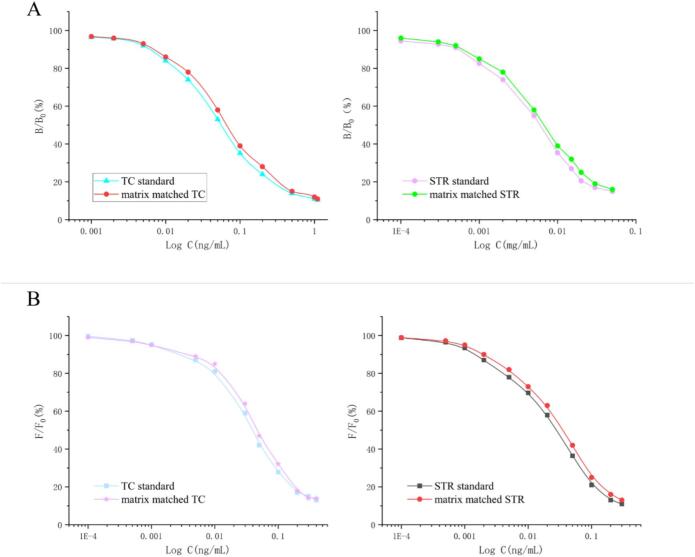
Competitive curves of (A) TC and STR standards and matrix's matched with TC and STR in multiplex direct competition chemiluminescence assay, and (B) TC and STR standards and matrix's matched with TC and STR in multiplex direct competition fluorescence assay.

Under optimal conditions, 10 tetracyclines and 12 aminoglycosides were prepared using extracts of blank pork samples and detected by multiplex direct competition chemiluminescence assay. As shown in Table 1, the IC_50_ values of the 12 AGs ranged from 3.6 to 17 ng/mL, with detection limits of 0.21–1.8 ng/mL. The IC_50_ values of the 10 TCs ranged from 0.0287 to 0.0427 ng/mL, with detection limits of 0.0016–0.0064 ng/mL. The representative competitive curves for TC and AG standards, as well as matrix-matched TC and AG, are shown in Figs. 6A, respectively. To assess recovery and variability, blank pork samples were fortified with different concentrations of the two drug classes for analysis. The recoveries of aminoglycosides were in the range of 68.8 %–92 %, with coefficients of variation ranging from 4.6 %–10.9 %. The recoveries of tetracyclines were in the range of 66 %–92 %, with coefficients of variation ranging from 4.6 %–8.8 % (Table 1).

**Table 1:** Sensitivity and recovery of 10 TCs and 12AGs from the standards fortified blank pork samples by multiplex direct competition chemiluminescence assay (*n* = 6).

Drug	IC_50_ (ng/mL)	LOD (ng/mL)	Added (ng/g)	Intra-assay		Inter-assay	
				Recovery (%)	CV (%)	Recovery (%)	CV (%)
STR	5.1	0.44	10	81.0	5.8	71.2	6.7
			100	85.8	5.3	77.3	5.2
AMK	5.1	0.48	10	87.3	6.5	81.3	7.9
			100	90.5	5.1	80.0	6.8
APM	5.9	0.68	10	91.0	5.3	82.7	6.0
			100	92.0	6.0	85.8	5.6
GEN	4.4	0.28	10	82.5	6.4	76.8	7.0
			100	85.3	5.2	78.5	5.9
NEO	4.8	0.38	10	74.8	8.1	70.3	10.9
			100	81.0	6.0	77.5	7.4
MIM	3.8	0.21	10	82.7	6.1	77.5	5.3
			100	85.3	5.2	78.3	5.5
SPM	9.8	0.9	10	77.2	6.1	74.5	5.5
			100	81.0	6.6	74.0	7.7
ETM	17	1.8	10	86.8	7.3	80.8	8.3
			100	91.3	6.9	85.7	8.6
NTM	16	0.79	10	79.7	6.5	80.0	6.3
			100	83.7	8.6	76.3	7.7
PMM	5.4	0.46	10	75.7	9.1	73.2	7.0
			100	80.8	7.4	78.0	10.6
ISM	3.6	0.22	10	81.8	9.6	80.0	7.0
			100	88.7	4.9	83.8	4.6
KAN	6.4	0.6	10	78.2	9.8	72.8	9.2
			100	79.3	7.4	68.8	8.3
DMC	0.0287	0.0022	10	74.7	6.7	66.0	6.8
			100	78.8	6.7	72.3	7.1
CTC	0.0344	0.0034	10	74.3	7.7	76.3	4.6
			100	78.5	5.9	74.8	6.3
OTC	0.0402	0.0028	10	84.0	4.9	78.0	7.0
			100	86.8	6.5	79.8	8.5
SC	0.0378	0.0016	10	72.7	6.4	77.2	6.5
			100	81.0	5.4	74.5	5.8
MTC	0.0418	0.0064	10	71.0	8.8	75.3	8.8
			100	75.8	5.7	75.5	6.1
MC	0.0357	0.0041	10	81.2	5.3	81.2	5.8
			100	81.3	5.1	80.0	6.6
TC	0.038	0.0047	10	78.3	6.7	77.3	7.5
			100	80.8	6.0	81.0	8.0
TIC	0.0414	0.0063	10	75.5	5.9	75.7	8.8
			100	83.7	7.1	85.3	6.9
LMC	0.0427	0.0053	10	83.8	7.6	80.8	5.2
			100	89.3	5.1	91.3	5.1
DC	0.0395	0.0057	10	89.3	7.0	81.3	5.8
			100	92.0	5.5	90.2	6.5

Under optimal conditions, 10 tetracyclines and 12 aminoglycosides were prepared using extracts of blank pork samples and detected by multiplex direct competition fluorescence assay. As shown in Table 2, the IC_50_ values of the 12 AGs ranged from 24.2 to 106.3 ng/mL, with detection limits of 1.1 to 10.2 ng/mL. The IC_50_ values of the 10 TCs ranged from 35.7 to 54.7 ng/mL, with detection limits of 2.7 to 6.4 ng/mL. The representative competitive curves for TC and AG standards, as well as matrix-matched TC and AG, are shown in Figs. 6B, respectively. To assess recovery and variability, blank pork samples were fortified with different concentrations of the two drug classes for analysis. The recoveries of aminoglycosides were in the range of 71.3 %–90 %, with coefficients of variation ranging from 5.1 %–9.8 %. The recoveries of tetracyclines were in the range of 68.7 %–91 %, with coefficients of variation ranging from 4.9 %–9.1 % (Table 2).

**Table 2:** Sensitivity and recovery of 10 TCs and 12AGs from the standards fortified blank pork samples by multiplex direct competition fluorescence assay (n = 6).

Drug	IC_50_ (ng/mL)	LOD (ng/mL)	Added (ng/g)	Intra-assay		Inter-assay	
				Recovery (%)	CV (%)	Recovery (%)	CV (%)
STR	27.3	1.8	10	82.5	5.8	77.3	6.0
			100	86.2	5.3	80.3	6.2
AMK	52.4	8.5	10	88.2	6.5	83.5	5.3
			100	90.0	5.1	81.8	5.4
APM	36.1	7.5	10	87.0	5.3	81.3	6.3
			100	86.3	6.0	87.8	5.8
GEN	27.7	1.7	10	85.5	6.4	80.5	6.7
			100	84.0	5.2	78.7	5.6
NEO	30.3	2.4	10	80.3	8.1	71.3	6.5
			100	80.7	6.0	79.5	5.7
MIM	26	1.4	10	79.8	6.1	79.2	5.5
			100	83.8	5.2	80.5	6.1
SPM	80.6	10.2	10	83.5	6.1	79.3	8.3
			100	87.3	6.6	77.8	7.9
ETM	106.3	9.2	10	86.7	7.3	81.7	5.3
			100	89.5	6.9	86.3	5.5
NTM	100.8	8	10	81.3	6.5	80.5	6.2
			100	85.7	8.6	78.5	5.7
PMM	33.4	5	10	79.8	9.1	74.3	7.4
			100	82.2	7.4	79.0	8.0
ISM	24.2	1.1	10	83.7	9.6	79.7	8.0
			100	86.0	4.9	84.3	6.9
KAN	33.9	5.5	10	79.2	9.8	76.2	6.7
			100	81.2	7.4	78.5	7.4
DMC	50.8	3.3	10	77.5	6.7	68.7	6.6
			100	82.7	6.7	74.2	5.5
CTC	54.7	2.7	10	78.0	7.7	78.5	5.9
			100	79.2	5.9	78.8	6.0
OTC	49.3	3.7	10	83.8	4.9	79.7	7.4
			100	86.2	6.5	78.8	6.2
SC	54	3.2	10	75.7	6.4	77.5	6.9
			100	83.7	5.4	83.5	7.8
MTC	41.8	6.4	10	77.2	8.8	79.5	8.1
			100	79.2	5.7	81.0	6.6
MC	35.7	4.1	10	82.8	5.3	83.2	6.6
			100	87.0	5.1	80.0	5.3
TC	38	4.7	10	78.8	6.7	79.3	7.4
			100	80.3	6.0	79.7	6.1
TIC	41.4	6.3	10	80.2	5.9	78.7	5.5
			100	85.0	7.1	88.5	5.0
LMC	42.7	5.3	10	81.8	7.6	81.7	5.4
			100	89.8	5.1	91.0	5.3
DC	39.5	5.7	10	87.5	7.0	82.8	5.3
			100	90.3	5.5	86.8	9.1

Based on our previous studies, the binding performance of the fusion protein was compared with that of the individual receptor (TetR). In the chemiluminescence method established using the individual TetR receptor, the LOD for ten tetracycline drugs ranged from 0.002 to 0.009 ng/mL, and the IC₅₀ ranged from 0.084 to 0.32 ng/mL. In contrast, the method developed with the fusion protein demonstrated improved sensitivity. The LOD for the same ten tetracyclines ranged from 0.0016 to 0.0064 ng/mL, while the IC₅₀ fell between 0.028 and 0.0427 ng/mL. The observed decreases in both LOD and IC₅₀ values across all tested tetracycline antibiotics indicate that the fusion protein exhibits slightly enhanced binding characteristics compared to the single receptor.

Finally, 50 pork samples were simultaneously analyzed using both the multiplex direct competition chemiluminescence method and the fluorescence method. In the multiplex direct competition chemiluminescence assay, two samples were identified as aminoglycoside-positive (15 ng/g and 19 ng/g, expressed as STR), while all others tested negative. The multiplex direct competition fluorescence method similarly detected two aminoglycoside-positive samples (12 ng/g and 14 ng/g, expressed as STR), with the remaining samples being negative. These findings indicate that the developed multiplex chemiluminescence and fluorescence methods can serve as effective rapid screening tools for high-throughput detection of aminoglycoside and tetracycline residues in pork samples. The extract samples from all 50 pork samples were also analyzed using our recently reported ultra-performance liquid chromatography (UPLC) method (Ma et al., 2020). The results confirmed that all samples were negative for tetracyclines. However, instrumental method capable of simultaneously detecting all 12 aminoglycosides remains under investigation. Therefore, the multiplex fluorescence and chemiluminescence methods developed in this study can serve as simple screening tools for the simultaneous detection of 10 tetracyclines and 12 aminoglycosides in large numbers of pork samples.

### 3.7 Comparison with the related methods

A comprehensive comparison of the developed methods with previously reported immunoassays for the detection of TCs and AGs is summarized in Table 3. This comparison highlights several definitive advantages of our multiplexed approach.

**Table 3:** Comparison with the previous assays for detection of AGs and TCs.

Recognition reagent	Method	Analyte	Assay time (from add sample)	LOD (ng/g)	Ref.
TetR	CL	5 TCs	40 min	0.005–0.016	(Wang et al., 2019)
DNA oligonucleotide of Teto	CL	1 drug	30 min	0.1	(Meyer et al., 2020)
TetR	CL	1 drug	15 min	6.33	(Kling et al., 2016)
Commercial Ab	CL	1 drug	60 min	9.4	(Luo et al., 2016)
MAb	LFIC	4 TCs	10 min	0.027–0.045	(Xu et al., 2020)
AGs Ab	CL	4 AGs	30 min	0.38–1.25	(Zeng et al., 2022)
Kanamycin mAb	ELISA	2 AGs	100 min	0.022–0.13	(Jiang et al., 2018)
Gentamicin IgY and Kanamycin IgY	FPIA	2 AGs	5 min	7 and 170	(Li et al., 2017)
–	MS	6TCs	–	0.06–0.09	(Guo et al., 2017)
RPSL_12_-TetR	FIA	12 AGs and 10TCs	30 min	1.1–10.2	This study
RPSL_12_-TetR	CLIA	12 AGs and 10TCs	40 min	0.0016–1.8	This study

The most salient feature of our work is the capability for simultaneous multi-class detection. As evidenced in Table 3, existing immunoassays are predominantly limited to the detection of a single drug (Kling et al., 2016; Luo et al., 2016) or multiple analytes within the same drug class (Wang, Zhang, et al., 2019; Xu et al., 2020; Zeng et al., 2022) at best. Notably, the high sensitivity of instrumental methods like mass spectrometry (MS) has been demonstrated, with studies showing LODs as low as 0.06–0.09 ng/g for six tetracyclines (Guo et al., 2022). However, these methods are typically limited to single-class detection and are hampered by the requirements for sophisticated equipment, extensive sample preparation, and longer analysis times. In contrast, the detection method developed in this study, based on the RPSL_12_-TetR fusion protein, enables the quantitative determination of 10 TCs and 12 AGs, which has not been previously achieved. This dramatically improves screening efficiency and reduces sample and reagent consumption.

Regarding sensitivity, the LODs of our chemiluminescence method (0.0016–1.8 ng/g) for TCs are comparable to or even lower than the most sensitive reported CL assays (Wang, Xia, et al., 2019). More importantly, the sensitivity achieved for all 22 analytes is well below the Maximum Residue Limits (MRLs) set by regulatory bodies, confirming their excellent suitability for regulatory screening. Furthermore, our methods exhibit superior operational efficiency without sacrificing sensitivity. The total assay time for both our fluorescence (30 min) and chemiluminescence (40 min) methods is highly competitive. It is significantly shorter than that of conventional ELISA protocols, which require incubation times exceeding one hour (Jiang et al., 2018b), and also compares favorably with the sample preparation and instrumental analysis time typically required by MS-based methods (Guo et al., 2022). Meanwhile the fluorescence polarization (FP) immunoassay (Li et al., 2017), despite its shorter incubation time, exhibits considerably higher LODs (7 and 170 ng/g) than our method for AGs detection. Finally, from both practical and economic perspectives, the use of fusion proteins demonstrates significant advantages over conventional antibody-based reagents. This strategy eliminates the requirement for animal immunization, thereby enabling sustainable, scalable, and cost-efficient production. Furthermore, it offers markedly improved batch-to-batch consistency compared to both polyclonal (pAb) and monoclonal (mAb) antibodies.

## 4 Conclusion

In this study, we successfully constructed a novel fusion protein, RPSL_12_-TetR, and developed two multiplex immunoassays for the simultaneous detection of 10 tetracyclines and 12 aminoglycosides in pork samples. This work is the first report of a dual-receptor fusion strategy applied to the simultaneous screening of two distinct classes of antibiotics. The multiplex chemiluminescence assay demonstrated ultra-high sensitivity with detection limits as low as 0.0016 ng/mL, while the multiplex fluorescence assay offered rapid and simplified operation, completing analysis within 35 min with a single incubation step. Both methods exhibited excellent recovery and precision in spiked pork samples, meeting regulatory screening requirements. The fusion protein approach not only eliminates the need for animal immunization but also provides a cost-effective and reproducible alternative to conventional antibodies. This study establishes a robust platform for high-throughput multi-class antibiotic screening and paves the way for developing novel biosensing strategies beyond traditional immunoassays.

## CRediT authorship contribution statement

**Wanqiu Xia:** Writing – original draft, Methodology. **Di Zhang:** Writing – original draft, Methodology. **Kuijing Liang:** Data curation. **Jiajia Hu:** Data curation. **Jiaxuan Chang:** Data curation. **Jianping Wang:** Writing – review & editing, Conceptualization.

## Declaration of competing interest

The authors declare that they have no known competing financial interests or personal relationships that could have appeared to influence the work reported in this paper.

## Data Availability

Data will be made available on request.
